# Two-step crystal growth mechanism during crystallization of an undercooled Ni_50_Al_50_ alloy

**DOI:** 10.1038/srep31062

**Published:** 2016-08-03

**Authors:** Simin An, Jiahao Li, Yang Li, Shunning Li, Qi Wang, Baixin Liu

**Affiliations:** 1 Key Laboratory of Advanced Materials (MOE), School of Materials Science and Engineering, Tsinghua University, Beijing 100084, China

## Abstract

Crystallization processes are always accompanied by the emergence of multiple intermediate states, of which the structures and transition dynamics are far from clarity, since it is difficult to experimentally observe the microscopic pathway. To insight the structural evolution and the crystallization dynamics, we perform large-scale molecular dynamics simulations to investigate the time-dependent crystallization behavior of the NiAl intermetallic upon rapid solidification. The simulation results reveal that the crystallization process occurs *via* a two-step growth mechanism, involving the formation of initial non-equilibrium long range order (NLRO) regions and of the subsequent equilibrium long range order (ELRO) regions. The formation of the NLRO regions makes the grains rather inhomogeneous, while the rearrangement of the NLRO regions into the ELRO regions makes the grains more ordered and compact. This two-step growth mechanism is actually controlled by the evolution of the coordination polyhedra, which are characterized predominantly by the transformation from five-fold symmetry to four-fold and six-fold symmetry. From liquids to NLRO and further to ELRO, the five-fold symmetry of these polyhedra gradually fades, and finally vanishes when B2 structure is distributed throughout the grain bulk. The energy decrease along the pathway further implies the reliability of the proposed crystallization processes.

Solidification processes of alloys have attracted increasing attention since they play critical roles in determining the microstructures which can exert significant influence on the physical and mechanical properties of the alloys. A rapid solidification process^1^, accompanied by a high crystallization rate, may lead to the formation of the metastable structures since the atoms attaching from the liquid to the solid may not have sufficient time to relax to the proper lattice sites. This process is interpreted as the disorder trapping in the literature^2^. The formed metastable structures, such as the dendrites^3^ and defects^4^ etc., could potentially affect the mechanical properties. Consequently, considerable attentions have been paid to characterizing the role of the metastable structures in the procedure of crystallization. Concerning this issue, Boettinger and Aziz^2^ have developed a comprehensive theory for the non-equilibrium crystallization kinetics of intermetallics. Along the development of this theory, researchers have conducted extensive experiments to identify clues of the metastable structures and their influence on solidification behaviors^5^,^6^,^7^. However, the structural rearrangements from metastable structures to the ordered state are on a rather short time scale^8^, so it is difficult to observe the details of the rearrangements through experiments, thus limiting further investigation of the metastable structures. Computational simulations with their high time resolution, however, have advantages on probing the nature of the non-equilibrium crystallization process^4^,^9^,^10^,^11^. For instance, through computational simulations, the effects of solute concentration on kinetics of the non-equilibrium crystallization process have been investigated widely^12^,^13^,^14^,^15^,^16^,^17^. Yet there are far fewer simulation studies focusing on the pathway of the metastable structural rearrangements during the non-equilibrium crystallization process of the intermetallic compounds^4^,^7^,^18^.

It is well established that the *B*2-ordered NiAl intermetallic with the highest melting temperature (about 1911 K)^19^ in the Ni-Al system can serve as good candidates for high-temperature structural materials. Its physical and mechanical properties combined with low density^20^,^21^,^22^,^23^ are important properties for technological applications, such as automotive, aeronautical and astronautical applications^24^,^25^. Nevertheless, the poor room temperature ductility of the NiAl intermetallic limits its industrial application. It has been considered that rapid solidification could be a promising way to overcome this limitation. Therefore, a systematic investigation of the rapid solidification involving the metastable structures would appear to be a demanding task.

In the present work, we investigate the crystallization process of the *B*2-ordered NiAl system by means of large-scale molecular dynamics simulations, and focus on the complex structural rearrangements originating from the metastable structures. In order to better illustrate the difference between the metastable structures and the “perfectly” crystallized structures, hereafter we employ the terms “non-equilibrium long rang order” (NLRO) and “equilibrium long rang order” (ELRO) to describe the regions assigned to either of both structures. The related two-step growth process is revealed by analyzing the structure and dynamics of the NLRO and ELRO regions. Besides, the interplays among the liquid, NLRO and ELRO regions would presumably dictate the crystallization process of this alloy.

## Results and Discussion

### Structural characterization

**Figure 1:**
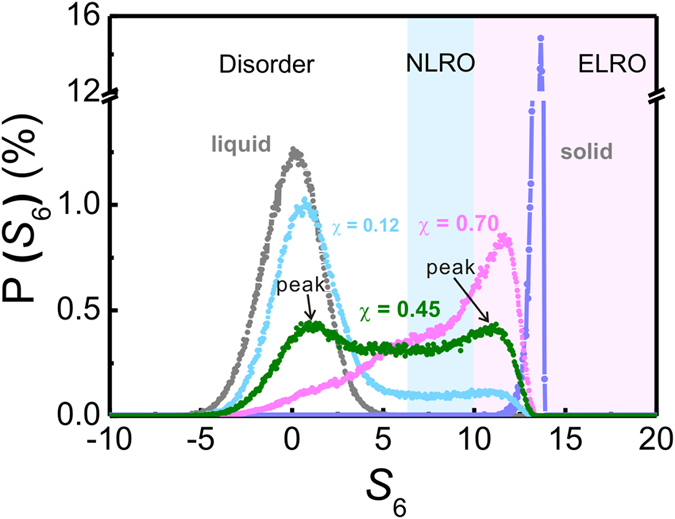
Distribution of structure order parameter *S*_*6*_ of the *B*2 NiAl system for perfect crystalline, liquid state and another three states with a fraction of crystalline atoms χ = 0.12, 0.45 and 0.70.

For the purpose of comprehending the relationship between *S*_6_ and the degree of order, we calculate the distribution of *S*_6_ in the *B*2 NiAl system for perfect crystalline, liquid state and another three states with the fraction of crystalline atoms χ = 0.12, 0.45 and 0.70 as shown in Fig. 1. Obviously, the high value of *S*_6_ implies the high degree of structural order. The *S*_6_ of the perfect crystalline NiAl intermetallic is predominantly fixed at 13 while the *S*_6_ of the liquid distributes symmetrically around zero stretching from −6 to 6. When χ = 0.45, the range of *S*_6_ exhibits two peaks in Fig. 1 representing the coexistence of liquid (left peak) and solid (right peak) atoms, respectively. Studies by Mütschele reported that the fraction of interfaces for grains with diameter of 5 nm can be as much as 50%^26^,^27^. These interfaces can be recognized as the metastable structures (or NLRO regions) as discussed above. To achieve the compatibility with this value, we set a threshold of 10 for *S*_6_, through which the NLRO regions that are denoted by 6.5 < *S*_6_ < 10 would amount to nearly a half of the crystallized regions (*S*_6_ > 6.5). In this sense, *S*_6_ = 10 is chosen as a criterion to distinguish between NLRO and ELRO regions in one grain. Similar standards were also employed in previous works^28^,^29^. Three distinct regions are displayed in Fig. 1: i) disordered region (*S*_6_ < 6.5); ii) NLRO regions (6.5 < *S*_6_ < 10); iii) ELRO regions (*S*_6_ > 10). We calculate Voronoi indices of the crystalline atoms with *S*_6_ > 10. The results, as shown in the Supplementary Fig. S1, indicate that about 90% of these crystalline atoms can be indexed to <0, 6, 0, 8>, which further validates the rationality of the criterion.

### Isothermal crystallization of the undercooled Ni_50_Al_50_ liquid

**Figure 2:**
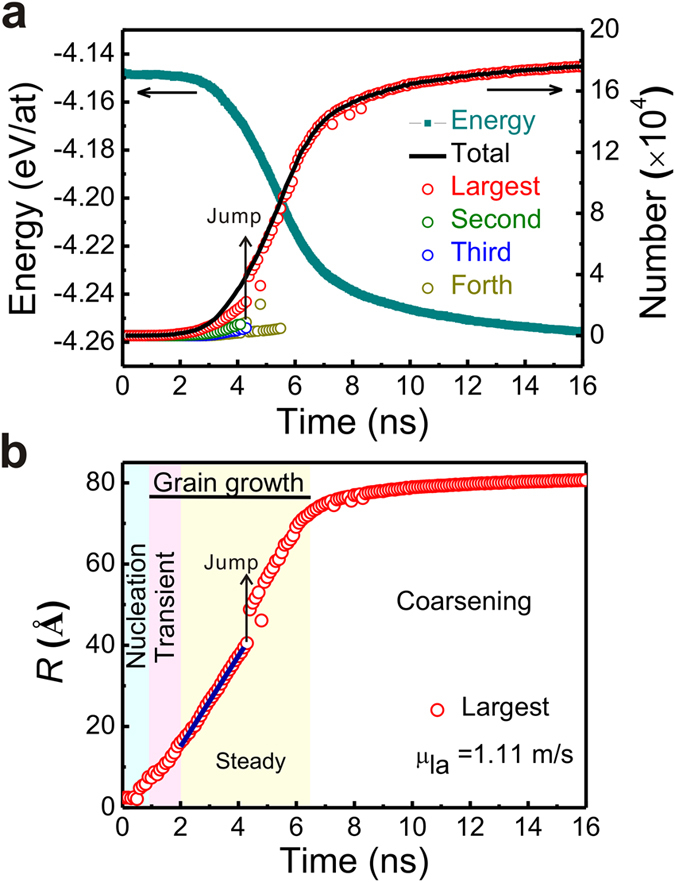
The time evolution of crystallization. (**a**) Potential energy and the number of crystalline atoms (N-T curve) in the whole system and (**b**) effective radius of the largest grain (R-T curve) of Ni_50_Al_50_ alloy annealing at 950 K. (**b**) The linear fit (blue lines) gives the growth rates of the largest grain. Note that the colors of the background are used to identify the different stages in the following figures.

Nucleation and subsequent crystal growth of Ni_50_Al_50_ alloy are observed by isothermal annealing of the simulation boxes at 900 K, 925 K, 950 K, 975 K, and 1000 K. Since nucleation is stochastic, the first nucleus appears after different induced time at different temperatures (see Supplementary Fig. S2). Above 975 K, only one nucleus grows throughout the simulation time, while below 950 K several nuclei form on hundreds of picoseconds. The merging and separating occur more and more frequently as the temperature decreases from 950 K to 900 K due to the increased nucleation rates. Here, we will take 950 K as a typical example to analyze the non-equilibrium crystallization process of Ni_50_Al_50_ alloys. The numbers of crystalline atoms and potential energy as a function of time are shown in Fig. 2a. The number of total crystalline atoms increases sharply from 2.5 ns to 8 ns accompanied by the dramatic decrease of energy, representing the large-scale crystallizing behavior. Before 4.3 ns, several grains grow simultaneously. At 4.3 ns, merging between the three largest grains occurs, which results in jumps in the growth curves of the largest grain at 4.3 ns in Fig. 2a,b. Atomic configurations before and after merging are depicted in the Supplementary Fig. S3. Subsequently, more than 90% of the total crystalline atoms belong to the new largest grain.

In order to distinguish the nucleation and crystal growth stage of the largest nucleus, we apply mean first-passage time (MFPT) method developed by Wedekind *et al*.^30^,^31^ to estimate the number of atoms in the critical embryo. The fitting result gives an estimation of 68 for the threshold. As a means of minimizing the effects of nucleation on investigations of the growth stage, the size of the critical embryo is defined as 110, which is comparable with other results derived from both experimental and theoretical work^5^,^27^,^32^. Therefore, growth stage of the largest grain is determined to begin at 0.9 ns as soon as the size of the grain exceed 110. Therefore, the three stages of crystallization for the largest grain are assigned as nucleation (before 0.9 ns), grain growth (0.9 to 6.5 ns) and coarsening (after 6.5 ns), as marked in effective radius of grains as a function of time (R-T curves, see Fig. 2b). The growth stage can be further partitioned into two characteristic stages, i.e. the transient growth (0.9 to 2 ns) and the steady growth (2 to 4.3 ns) stages, according to different growth mechanisms. The largest grain grows linearly at the steady growth stage with the rate of 1.11 m/s obtained by linear fitting of the R-T curves. The steady growth rate has the same order of magnitude with the experimental measurements of NiAl non-equilibrium growth rates^3^,^7^,^33^, which demonstrates the reliability of our calculation results (Fig. 2b).

**Figure 3:**
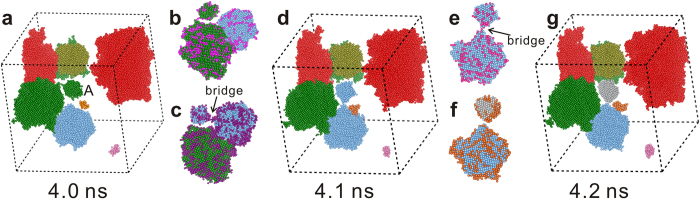
Several representative snapshots at different time for merging and separating process of grains. The red, green and light blue clusters represent the largest, second largest and the third largest grains, respectively. The rose balls in (**b**) and the pink balls in (**e**) represent the loss of atoms at 4.0 ns and 4.1 ns respectively. The purple in (**c**) and the orange in (**f**) represent the gain of atoms at 4.0 ns and 4.1 ns, respectively.

Except for the obvious merging at 4.3 ns mentioned previously, there is also hidden merging before 4.3 ns. Frequent merging and separating between different grains directly reflect the tendency that the metastable NLRO atoms are rearranged to the ordered state. Figure 3 illustrates this process among the second largest grain (green cluster), the third largest grain (light blue cluster) and the smaller grain (cluster-A) through only a few metastable bridge atoms. Cluster-A solely attaches to the second largest grain at 4.0 ns. However, it transfers to the third largest grain at 4.1 ns. Afterwards, it departs from the third largest grain and becomes an isolated grain. This fast transformation process implies that NLRO atoms on the surface of the grains are metastable and likely to diffuse to the proper lattice sites^3^. To describe this process in detail, the gain and loss of atoms in these grains were analyzed. Some of the NLRO atoms (colored in rose in Fig. 3b) including bridge atoms at 4.0 ns transform into liquid state at 4.1 ns, resulting in the separation between the second largest grain and the cluster-A. Meanwhile, some liquid atoms would attach to the surface (colored in purple in Fig. 3c). These attached atoms not only contribute to the growth of these grains, but also serve as a bridge between the third largest grain and the cluster-A. Subsequently, bridge atoms at 4.1 ns turn into liquid state at 4.2 ns (Fig. 3e) and no new bridge atoms is generated afterwards (Fig. 3f). The transformation between these grains will end when sufficient bridge atoms are rearranged to proper lattice sites. Note that during the transformation, the metastable surface atoms with NLRO are relaxed to a steady state with ELRO. It can be concluded that the transitions from the liquids to the perfect crystals occur involving the initial formation of the metastable NLRO regions which could be rearranged to ELRO regions to form perfect crystals. This exhibits an interesting two-step growth pathway: liquid → metastable NLRO regions → steady ELRO regions. The second step is generally regarded as structural rearrangements. Hereafter, we will focus on the growth stage of the largest grain to display the details of two-step growth mechanism.

### The appearances of structural rearrangements

**Figure 4:**
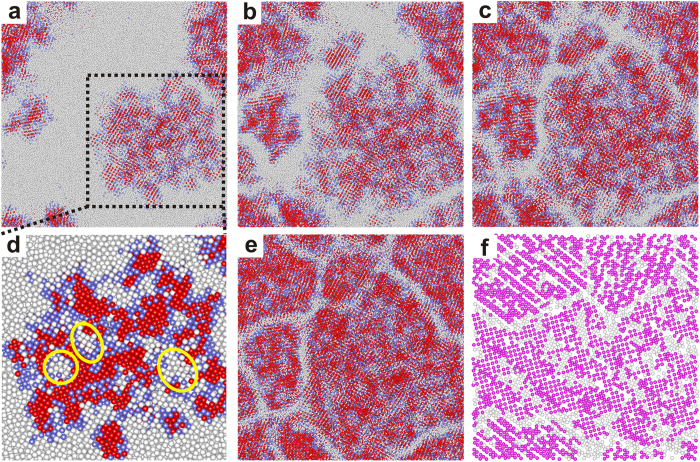
Projections from the cross section of the simulation box at (**a**) 3.9 ns, (**b**) 5.1 ns, (**c**) 6.1 ns (**d**) magnified grain at 3.9 ns and (**e**) 16.0 ns. The white, blue and red balls stands for atoms distributed in the disorder regions, NLRO regions and ELRO regions, respectively. (**f**) A snapshot of a slice of the simulation box at 16 ns, showing the configuration inside the simulation box. The purple balls represent the sum of the NLRO and ELRO atoms.

Figure 4 illustrates the process of structural rearrangements through the atomic projections from the cross section of the simulation box. Notably, the largest grain exhibits an inhomogeneous structure. Metastable NLRO regions (colored in blue) are distributed not only at the interfaces but also inside the grains. Usually, these NLRO atoms inside the grains enclose a group of disordered atoms to form bulk defects, which are marked by the yellow circles in Fig. 4d. With respect to the formation of NLRO regions and bulk defects inside the grain, it is inferred that the rearrangement of the newly-formed NLRO atoms may be slower than the growth of the grain. Thus these NLRO atoms will be encapsulated inside the grain and some of them even transform into a disorder state later on. It is obvious that the bulk defects (white regions inside the grain) shrink as accompanied by grain growth with prolonged relaxation time (Fig. 4a–c). Using an image analysis software – ImageJ^34^, we measure the area fractions of the bulk defects at the growth stage. With apparent variations between images taken at 3.9 ns, 5.1 ns and 6.1 ns, the area fractions turn out to be 14.46%, 12.32% and 8.57% respectively. Hence the atoms attached to the grains would have NLRO at first and then are rearranged to ELRO when the grains grow larger. Finally in Fig. 4e, the grains tend to transform into a much more ordered structure and the grain boundaries become much clearer. It is observed in Fig. 4f that an ordered crystal has formed with vacancies stemming from structural rearrangements.

### Kinetics and structural evolution of two-step growth mechanism

**Figure 5:**
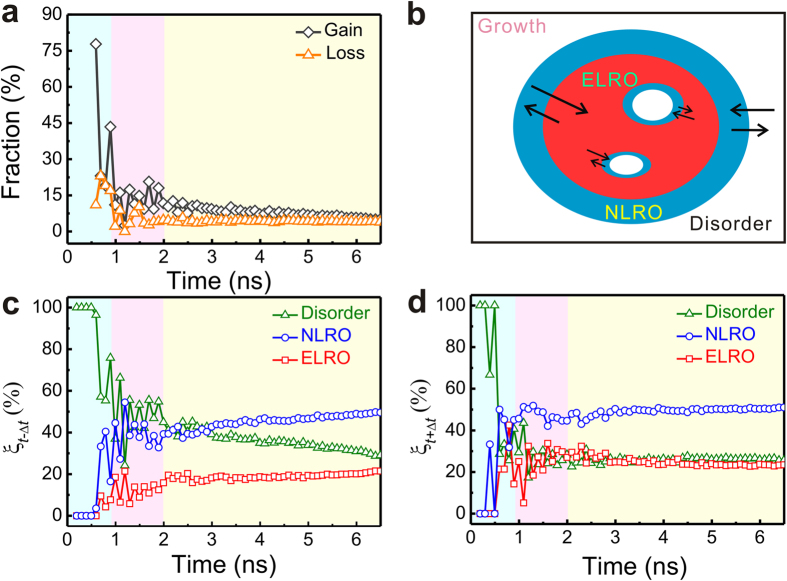
(**a**) The fraction of the ELRO atoms gain (black curves) and loss (orange curves) from the liquid atoms. (**b**) A schematic of structural rearrangements. The longer the arrow is, the more atoms are rearranged into the region that the arrow points to. (**c**,**d**) Time evolution of the fraction of the NLRO atoms (**c**) gain (*ξ*_*t*−Δ*t*_) and (**d**) loss (*ξ*_*t*+Δ*t*_) from ELRO, NLRO and disordered regions in the largest grain. The backgrounds in (**a**,**c**,**d**) are consistent with Fig. 2b.

In order to get more insight into the mechanism of the two-step growth process, we trace the variations of structural order parameter of atoms as shown in Fig. 5. (Supplementary Fig. S4 shows the variations throughout the whole simulation time) First, the exchange of atoms between ELRO regions and liquids in the largest grain is investigated and exhibited in Fig. 5a. Hereafter, unless otherwise specified, the “gain” refers to the atoms that are derived from any of the three regions and the “loss” refers to the atoms that transfer out into any of the three regions. Among the ELRO atoms, the fraction of gain from the liquids is around 15% at the transient growth stage since the newborn grain is small along with high specific surface area. At the steady growth stage, this faction steadily decreases accompanied by the growth of the grain. It is observed that the fractions for both gain and loss of the ELRO atoms from the liquids are lower than 10% near the end of the steady growth stage, indicating that it is difficult for liquids to directly crystallize into perfect crystal. On this occasion, NLRO regions play a critical transition role on the two-step growth process. Therefore, we focus on the structural rearrangements of NLRO regions, as illustrated by the schematic in Fig. 5b.

Considering the period of (*t* − Δ*t*) → *t*, these NLRO atoms are derived from the disordered regions and the ELRO regions, contributing to the gain of NLRO atoms. Considering the period of *t* → (*t* + Δ*t*), part of NLRO atoms transform into the ELRO atoms, making the grains more ordered, and some other NLRO atoms turn to be disordered atoms, contributing to the loss of NLRO atoms. In order to describe the above two processes and characterize the structural rearrangements kinetics, two fractions are defined as follows:


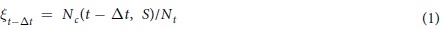



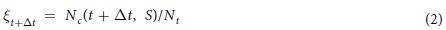


where *N*(*t*) is the number of NLRO atoms at the time *t* and *N*_*c*_ (*t* − Δ*t*, *S*) represents the number of these NLRO atoms which belonged to the *S* region, *i.e.* the disordered, NLRO or ELRO regions, at the time (*t* − Δ*t*). In other words, the *ξ*_*t*−Δ*t*_ denotes the ratio of the number of NLRO atoms derived from the *S* region to that of the total NLRO atoms at the time *t* and *ξ*_*t*+Δ*t*_ denotes the ratio of the number of NLRO atoms that will transform into the *S* region at the time (*t* + Δ*t*) to that of the total NLRO atoms at the time *t*. The results is demonstrated in Fig. 5c,d. At the initial nucleation stage, NLRO atoms form the nucleus, and the atomic exchange occurs between the nucleus and the liquids due to the large surface area of the nucleus. At the end of the nucleation stage, the ELRO regions form. The fractions of NLRO atoms transforming into ELRO regions (*ξ*_*t*+Δ*t*_ for ELRO regions) are much higher than that derived from ELRO regions (*ξ*_*t*−Δ*t*_ for disordered regions), indicating that NLRO regions are rearranging themselves more orderly. At the growth stage, the fractions of NLRO atoms derived from disordered regions (*ξ*_*t*−Δ*t*_ for disordered regions) are much higher than that transforming into disordered regions (*ξ*_*t*+Δ*t*_ for disordered regions), especially at the transient growth stage. The gain of NLRO atoms from disordered regions is as high as 25%. Yet *ξ*_*t*+Δ*t*_ for NLRO regions is slightly higher than *ξ*_*t*−Δ*t*_ for NLRO regions, about 7%, indicating an extra fraction of NLRO atoms that must transform into other regions. Correspondingly, in comparison with *ξ*_*t*−Δ*t*_ for ELRO regions, an excess fraction is found by *ξ*_*t*+Δ*t*_ for ELRO regions, reconfirming the transformation from NLRO regions into ELRO regions. Moreover, the variations of structural order parameter at other temperatures display very similar features, which further confirms the reliability of the two-step growth pathway (see Supplementary Fig. S5).

**Figure 6:**
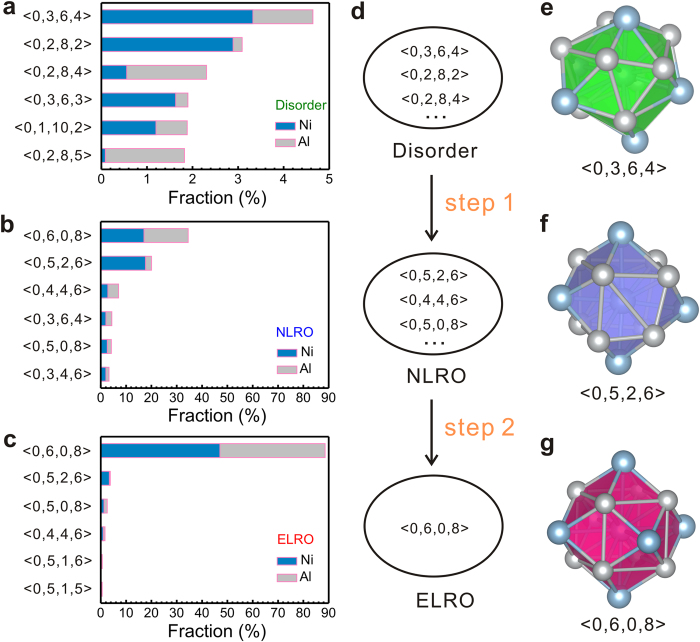
The population of the six most populous Voronoi polyhedra in the (**a**) disordered regions, (**b**) NLRO regions, and (**c**) ELRO regions. The dark blue and gray bars represent Ni- and Al-centered Voronoi polyhedra respectively. (**d**) The schematic of two-step growth mechanism. The typical Voronoi polyhedra in the (**e**) disordered regions, <0, 3, 6, 4>, (**f**) NLRO regions, <0, 5, 2, 6>, and (**g**) ELRO regions, <0, 6, 0, 8>.

**Figure 7:**
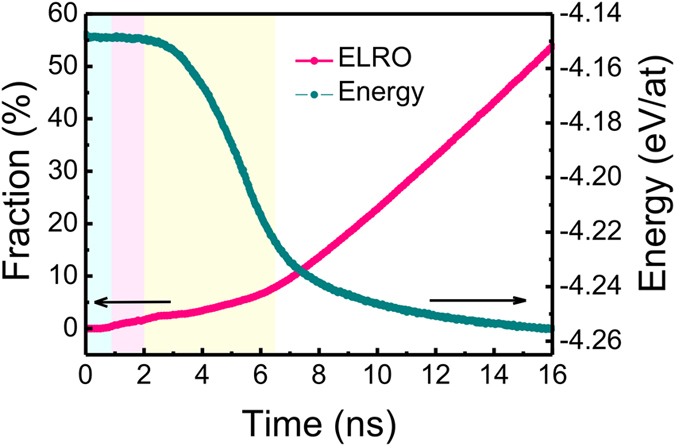
The fraction of ELRO atoms in the total crystalline atoms (the sum of ELRO atoms and NLRO atoms) and the potential energy of the whole system as a function of time. The potential energy is the one that has been shown in the Fig. 2.

To illustrate the evolution of the primary structure at the two-step growth pathway, the populations of Voronoi polyhedra at different times are analyzed. The populations of Voronoi polyhedra at 3.9 ns, 4.1 ns, 4.3 ns, and 4.5 ns are averaged to characterize the typical structural evolution in Fig. 6a–c. For disordered regions, the Voronoi polyhedra are complex and diverse. 5-edged face dominates in all the six most populous polyhedra. For NLRO regions, the two most populous polyhedra have Voronoi indices <0, 6, 0, 8> and <0, 5, 2, 6> with fractions less than 35% and their 5-edged faces are much less than the polyhedra in the disordered regions. For ELRO regions, more than 90% of the polyhedra have Voronoi indices <0, 6, 0, 8>, indicating that polyhedron <0, 6, 0, 8> is the dominant structure unit. In comparison with the ELRO region, the status of the polyhedron <0, 6, 0, 8> for NLRO regions is less evident. To further identify this relationship between the microscopic structure and the two-step growth, we also calculate the degree of local five-fold symmetry (LFFS) which is defined by the ratio of *n*_5_ to the sum of *n*_3_, *n*_4_, *n*_5_ and *n*_6_ in the Voronoi indices^35^,^36^. The structural changes reflect by LFFS are prominent. For body-centered cubic (BCC), the degree of LFFS is 0 corresponding to the Voronoi indices <0, 6, 0, 8>. For icosahedral polyhedron, the degree is 1 corresponding to the Voronoi indices <0, 0, 12, 0>. In the present work, it is shown that the degree is 0.465, 0.140 and 0.016 respectively in the disordered, NLRO and ELRO regions at 5.1 ns. With five-fold symmetry less prominent than the disordered regions and more prominent than the ELRO regions, the NLRO regions play an evident transition role. For Al, the populations of transition polyhedra in the NLRO regions, such as <0, 5, 2, 6>, <0, 4, 4, 6>, <0, 5, 0, 8>, are nearly the same (Fig. 6b) and the diversity of the polyhedra is remarkable. In contrast, for Ni, in the NLRO regions the number of polyhedra <0, 5, 2, 6> even exceeds that of <0, 6, 0, 8>. Moreover, in the ELRO regions polyhedra <0, 6, 0, 8> are the dominant ones and the polyhedra <0, 5, 2, 6> for Ni are far more than that for Al. This indicates that it is difficult for atom Ni to directly locate on the equilibrium lattice sites and the primary transition structure is polyhedra <0, 5, 2, 6>. We have traced the evolution of a series of Ni-centered polyhedra. It is detected that, during crystallization, a group of Ni-centered liquid atoms first build the transition structures like polyhedra <0, 5, 2, 6>, <0, 4, 4, 6>, <0, 5, 0, 8> etc. and then it will be easy for these atoms to construct polyhedra <0, 6, 0, 8>. Combined with the difficulties of the transformation from disordered regions to ELRO regions as discussed before (Fig. 5a), it can be inferred that <0, 5, 2, 6> are the foremost structure units of the NLRO regions. Figure 6e–g display the typical evolution of Ni-centered polyhedron from <0, 3, 6, 4> to <0, 5, 2, 6> and further to <0, 6, 0, 8>, which represent the disordered, NLRO and ELRO regions, respectively. The polyhedra <0, 3, 6, 4> and <0, 5, 2, 6> both consist of five Ni atoms and eight Al atoms. The 13 atoms in the polyhedron <0, 3, 6, 4> arrange with very low periodicity. The polyhedron <0, 3, 6, 4> has six 5-edged faces, exhibiting significant five-fold symmetry which is sensitive to disordered structures. After the short-range diffusions of the 13 atoms in the polyhedron <0, 3, 6, 4>, it transforms into a relatively high-symmetric polyhedron <0, 5, 2, 6> with only two 5-edged faces. Subsequently, the first step of grain growth has completed, corresponding to the transformation from disordered regions to the NLRO regions. It is observed that <0, 5, 2, 6> is actually the polyhedron <0, 6, 0, 8> with a missing Ni-atom at the specific site. Adding the Al-atom, <0, 5, 2, 6> transforms into polyhedron <0, 6, 0, 8>, exhibiting the perfect symmetry of *B*2 structure and then the second step of grain growth from NLRO regions to ELRO regions has completed. The populations of Voronoi polyhedra at other temperatures show almost the same results (see Supplementary Fig. S6). Figure 7 displays the relationship between the structures and the energy. At the nucleation stage and the transient growth stage, the disordered atoms constitute more than 99% of the system and the potential energy of the system is the highest. At the coarsening stage, the number of the crystalline atoms reaches the maximum as shown in Fig. 2a, while the fraction of the ELRO atoms increase along with the decrease of the energy as shown in Fig. 7. This indicates that disordered, NLRO and ELRO regions own the highest, medium and lowest energy, respectively. Provably, the corresponding characteristic energy of the three regions with the feature structures are about −4.14, −4.30 and −4.36 eV/atom, respectively. Combined with the feature of the Voronoi polyhedra discussed before, the potential energy reduces with the structural transformations from five-fold symmetry to four-fold and six-fold symmetry. As a result, the two-step growth mechanism allows the system following the pathway with the decreased energy. In conclusion, two-step grain growth mechanism is a significant kinetic pathway for crystallization, since it is very difficult for the structural transformation from a low-symmetric polyhedron with highest potential energy straightly to a high-symmetric polyhedron with lowest potential energy. On this occasion, the formation of the transition characteristic polyhedra promotes the crystallization process by decreasing the resistance for crystal growth.

## Conclusion

Using large-scale MD simulations, the time-dependent non-equilibrium crystallization behavior of the NiAl alloy were investigated. We have observed the kinetics of the crystallization which obviously reveals a two-step growth mechanism: from liquid to metastable NLRO regions and further to steady ELRO regions. With the very high growth rates during rapid solidification, the crystallization of liquids generally involves the initial formation of the metastable NLRO atoms. These metastable atoms arise both at the interfaces and inside the grains, making the grains inhomogeneous. Compared with disordered atoms, these NLRO atoms could be easier to transform to ELRO ones. We analyze the two-step growth mechanism. The foremost Ni-centered structural unit in the NLRO regions at this pathway is polyhedron <0, 5, 2, 6> in which the amount of 5-edged faces is fewer than that of those polyhedra in the ELRO regions. More than six 5-edged faces of the polyhedra in the ELRO regions are common. The dominant polyhedron in the ELRO regions has the indices <0, 6, 0, 8>, which is a standard structure for body-centered cubic. The structures of the polyhedra in the NLRO regions which play great roles of transitions are usually relevant to that in the liquids and that of the polyhedra <0, 6, 0, 8>.

## Methods

The MD simulations were implemented by using Large-scale Atomic/Molecular Massively Parallel Simulator (LAMMPS) packages^37^. The widely used embedded-atom method (EAM) potential elaborated by Mishin *et al*.^38^ was adopted to describe the interatomic interaction in the Ni-Al system. The melting point of bulk *B*2 NiAl intermetallic with the EAM potential was estimated at about 1520 K by Kerrache *et al*.^39^. There were 250,000 atoms in the initial B2 NiAl simulation box with dimensions of 14.3 nm × 14.3 nm × 14.3 nm. Periodic boundary conditions were applied in three spatial directions. The *NPT* ensemble was used throughout the simulations, in which the pressure (0 Pa) and temperature were controlled by single Nose–Hoover barostat and thermostat, respectively. The equation of motion were solved by using the Velocity Verlet algorithm with time-step of 2 fs. To simulate the rapid solidification process, the Ni_50_Al_50_ melts were first thermalized isothermally above the melting point for 4 ns to reach an equilibrium melts, and then quenched down to 950 K at the rate of 5.33 × 10^11^ K/s. Afterwards, the quenched alloys were annealed for 16 ns at the given temperatures. The time evolution of pressure and temperature during annealing are shown in Supplementary Fig. S7. Besides, to analyze the statistics of the two-step growth mechanism, the equilibrium melts were also quenched down to 900 K, 925 K, 975 K and 1000 K at the same rate and then annealed.

To monitor the structural evolution upon quenching and annealing of the Ni_50_Al_50_ alloys, two types of analyses were performed, i.e., the structural order parameter *S*_6_ and Voronoi index. The structural order parameter *S*_6_ of an atom is obtained as follows^40^,^41^,^42^


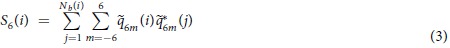



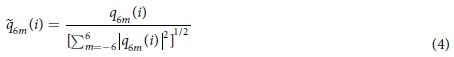



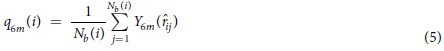


where 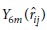 are the spherical harmonics defined by the vector 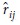 linking atom *i* and *j*. The atoms are considered as coordinating ones if their distances to the central atom are less than 3.6 Å, and *N*_b_ is coordination number of a central atom. In order to take account of the crystalline atoms at the liquid-solid interface, we define an atom as crystalline if it is surrounded by more than 13 neighbors as well as *S*_6_ > 6.5 (Fig. 1)^42^,^43^. The crystalline atoms constitute the grains that nucleate and grow during the solidification. To distinguish between individual grains and further estimate the grain size, two grains are considered to be disconnected if none of the crystalline atoms in one grain has neighboring atoms that belongs to another.

Voronoi index <*n*_3_, *n*_4_, *n*_5_, *n*_6_> is used in the present work to describe the local structures around the atoms. Here, *n*_*i*_ denotes the number of *i*-edged faces of the Voronoi polyhedra. For instance, <0, 6, 0, 8> represents a body-centered cubic structure, in which the central atom connects 14 neighboring atoms, and its Voronoi polyhedron is enveloped by six squares and eight hexagons^43^. The Voronoi polyhedra are also applied to track the evolution of the crystal structure during crystallization. In addition, we employ the Voronoi tessellation technique to measure the volume of a grain *V*_grain_ which is calculated by summing up the atomic Voronoi volumes *V*_atom_ in the grain. Subsequently, we calculate the effective radius of an individual grain as


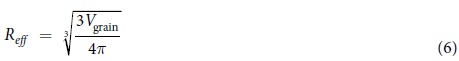


## Additional Information

**How to cite this article**: An, S. *et al*. Two-step crystal growth mechanism during crystallization of an undercooled Ni_50_Al_50_ alloy. *Sci. Rep.* **6**, 31062; doi: 10.1038/srep31062 (2016).

## Supplementary Material

Supplementary Information
