# Supplementary material for: Two-step crystal growth mechanism during crystallization of an undercooled Ni50Al50 alloy
Source: Sci Rep. 2016 Aug 3;6:31062. doi: 10.1038/srep31062 (PMC4971477; doi:10.1038/srep31062)
Supplement: Supplementary Information [file srep31062-s1.pdf]

Supporting information

Two-step crystal growth mechanism during crystallization of an undercooled $Ni_{50}Al_{50}$ alloy

Simin An, Jiahao Li, Yang Li, Shunning Li, Qi Wang and Baixin Liu

Key Laboratory of Advanced Materials (MOE), School of Materials Science and Engineering, Tsinghua University, Beijing 100084, China.

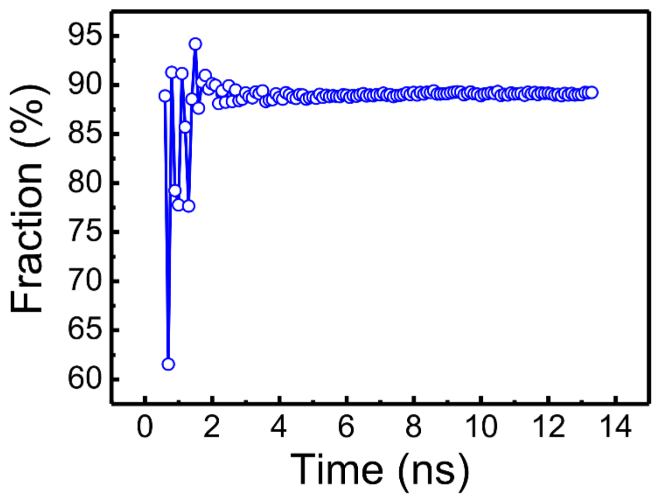

Figure S1. The fraction of the crystalline atoms with $S_6 > 10$ which can be indexed to $\langle 0,6,0,8 \rangle$.

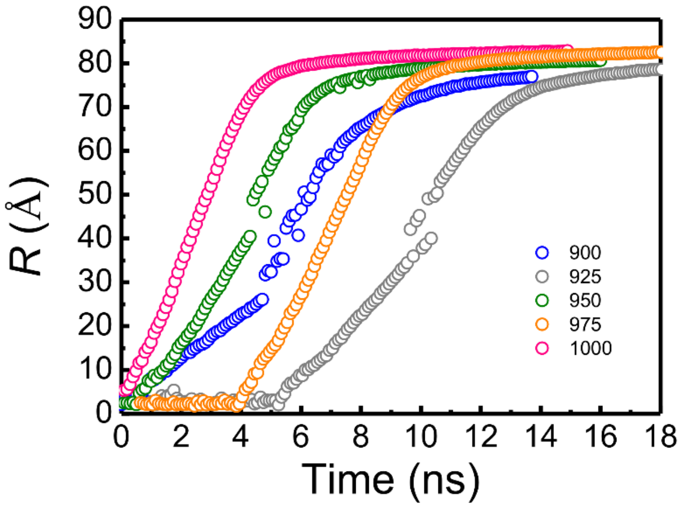

Figure S2. The time evolution of the effective radius of the largest grain (R-T curve) of $Ni_{50}Al_{50}$ alloy annealing at 900 K, 925 K, 975 K and 1000 K. Since nucleation is a stochastic process, the fluctuations of the induced time for nucleation may lead to the irregular of the R-T curves as the increase of the temperature.

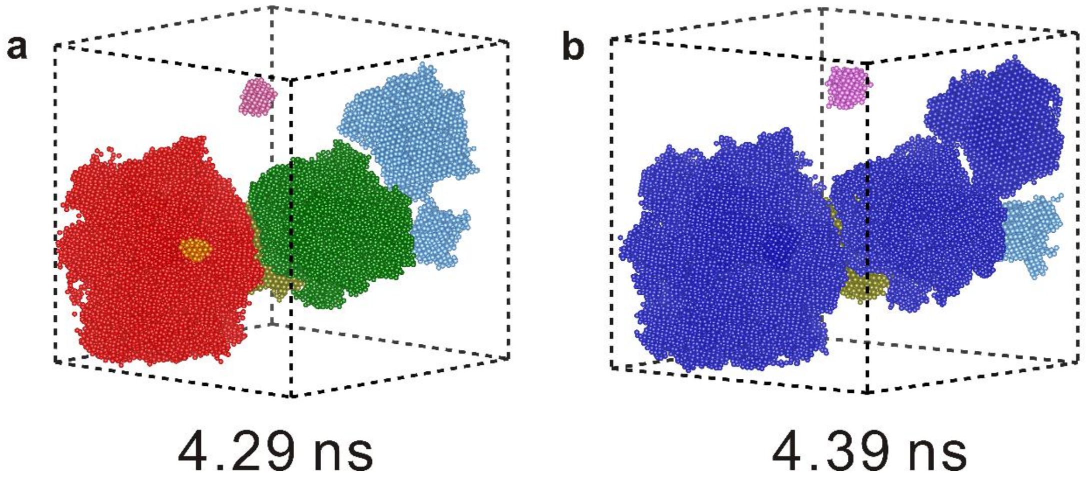

Figure S3. Snapshots of the merging between the three largest nuclei around 4.29 ns. The red, green and light blue clusters represent the largest, second largest and the third largest crystalline grains respectively at 4.29 ns. The dark blue cluster represents the largest nuclei after merging.

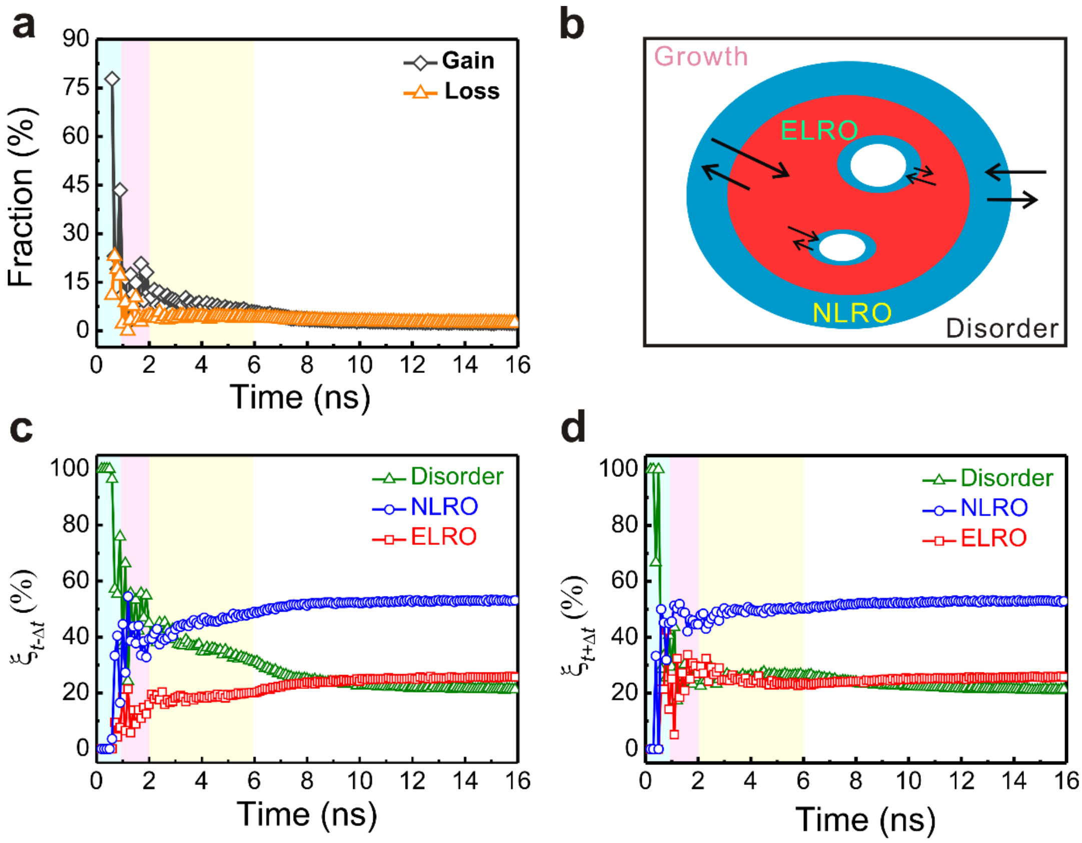

Figure S4. (a) The fraction of gain (black curves) and loss (orange curves) among the ELRO atoms from the liquid atoms. (b) A schematic of structural rearrangements. The longer the arrow is, the more atoms are rearranged into the region that the arrow points to. (c, d) Time evolution of the fraction of NLRO atoms (c) gain ($\xi_{t-\Delta t}$) and (d) loss ($\xi_{t+\Delta t}$) from ELRO, NLRO and disordered regions in the largest grain. The backgrounds in (a)(c)(d) are consistent with Fig. 2b, in which the light blue, light pink and light yellow respectively represents the nucleation stage, the transient growth stage and the steady growth stage. Figure S4 shows the time evolution of whole simulation of Figure 5 in the main text. During the nucleation time, the ξs in (c) and (d) fluctuate, indicating that nucleation is a stochastic process. After 6 ns, the fractions in (a) and ξs in (c) and (d) tend to be steady.

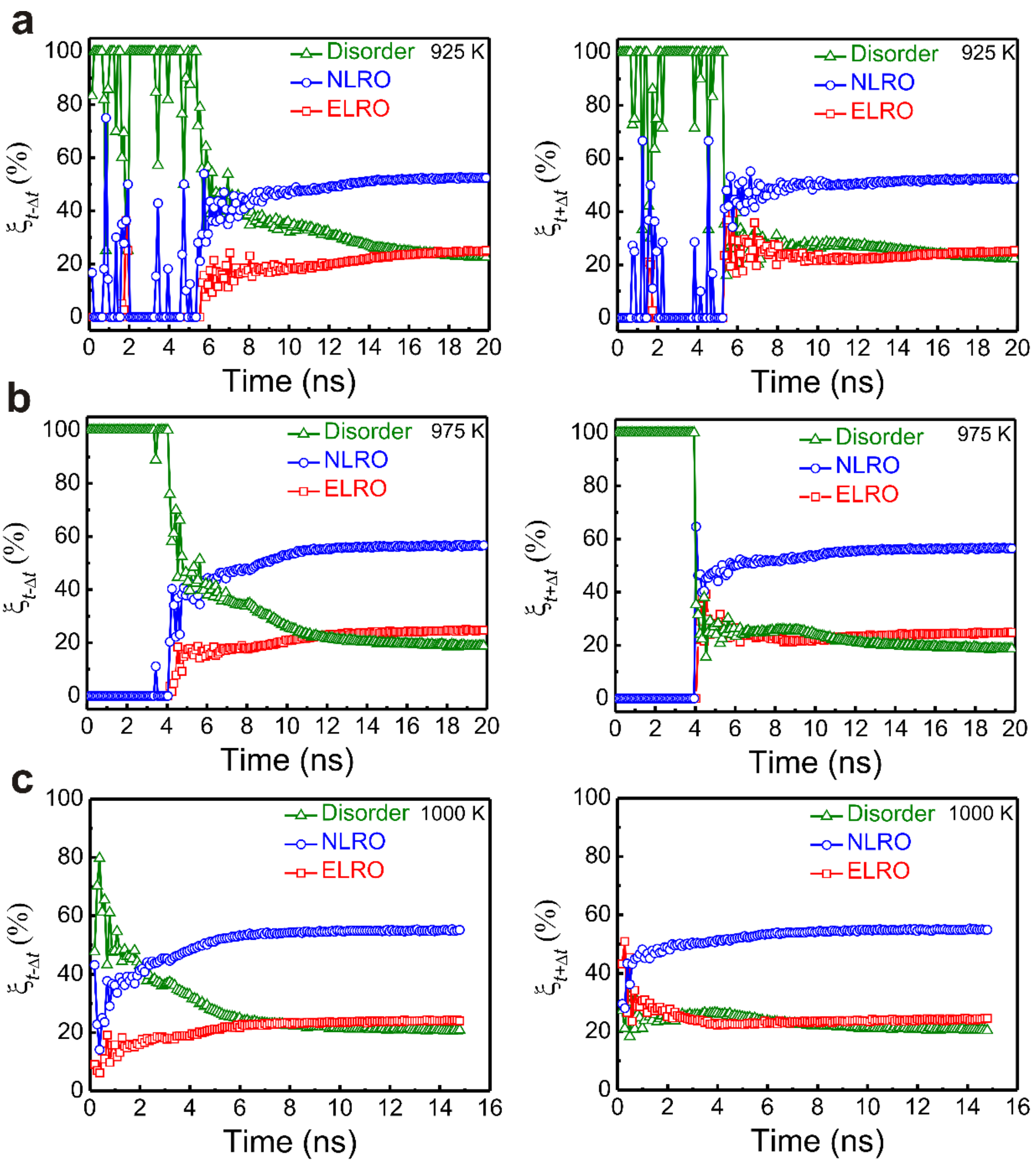

Figure S5. Time evolution of the fraction of NLRO atoms gained ($\xi_{t-\Delta t}$) and lost ($\xi_{t+\Delta t}$) from ELRO, NLRO and disordered regions in the largest grain at (a) 925 K, (b) 975 K and (c) 1000 K. During the nucleation time, the ξs fluctuate frequently and drastically at low temperatures while at high temperatures the fluctuations are rare due to the decrease of the nucleation rates when approaching the melting points.

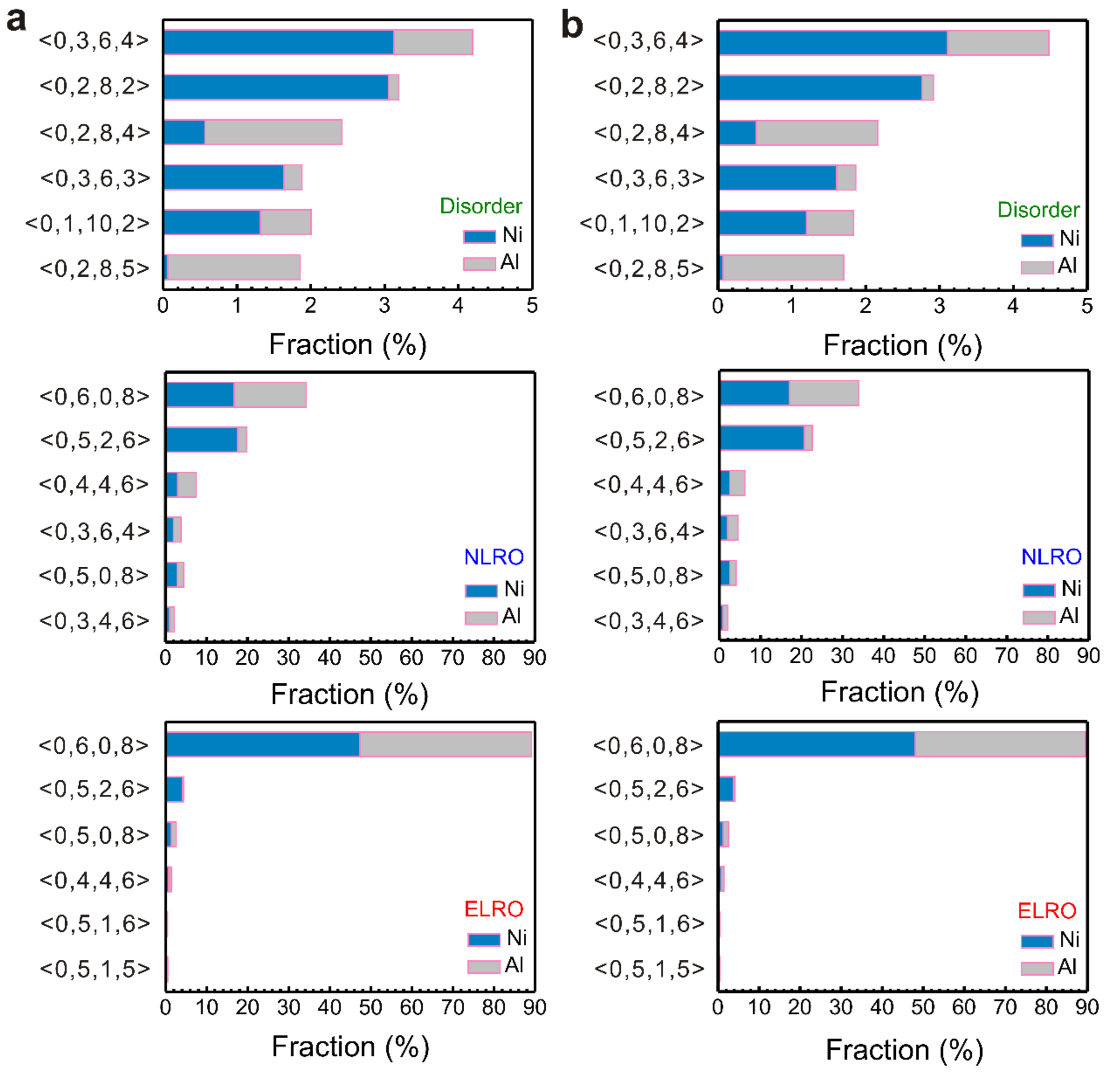

Figure S6. The averaged population of the six most populous Voronoi polyhedra in the disordered, NLRO, and ELRO regions at (a) 925 K (the average of 8.4 ns, 8.6 ns, 8.8 ns and 9.0 ns) and (b) 1000 K (the average of 2.6 ns, 2.8 ns, 3.0 ns and 3.2 ns). The dark blue and gray bars represent Ni- and Al-centered Voronoi polyhedra respectively.

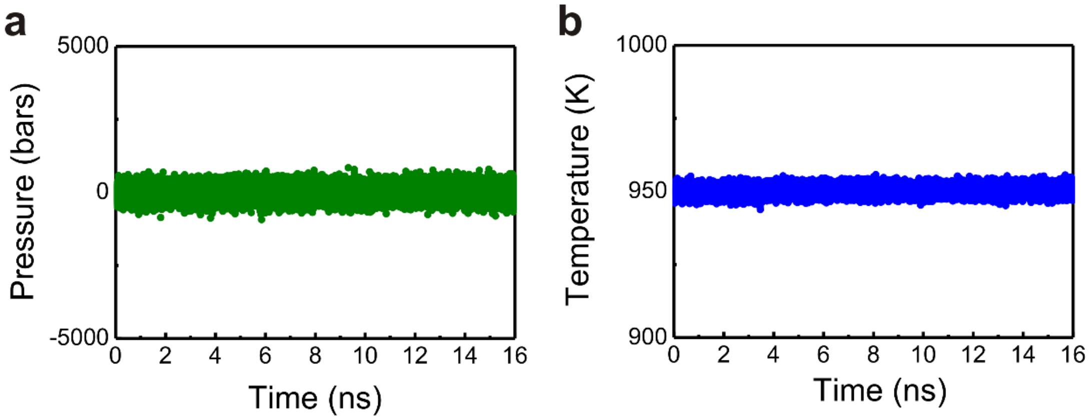

Figure S7. The time evolution of (a) pressure and (b) temperature during the annealing of the quenched alloys at 950 K. The pressure and temperature show a few fluctuations but remain stable around 0 bars and 950 K, respectively.

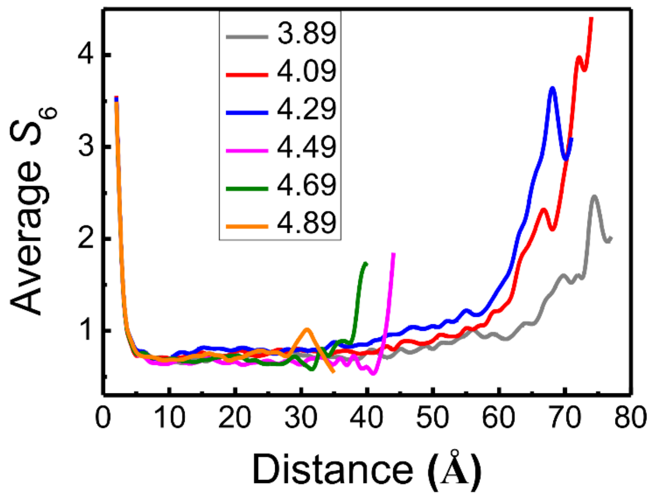

Figure S8. The average S_6 as a function of distance from the largest nucleus. Meeting other nuclei at different places makes the curves up asynchronously when the distance is longer than 35 Å. The gradual increases identify transitions from the liquids to the interfaces of the crystal.
